# Multiple rather than specific autoantibodies were identified in irritable bowel syndrome with HuProt™ proteome microarray

**DOI:** 10.3389/fphys.2022.1010069

**Published:** 2022-10-03

**Authors:** Wenjuan Fan, Xiucai Fang, Chaojun Hu, Guijun Fei, Qiyun Xiao, Yongzhe Li, Xiaoqing Li, Jackie D. Wood, Xuan Zhang

**Affiliations:** ^1^ Department of Gastroenterology, Peking Union Medical College Hospital, Chinese Academy of Medical Sciences and Peking Union Medical College, Beijing, China; ^2^ Department of Gastroenterology, Tongji Hospital, Tongji Medical College, Huazhong University of Science and Technology, Wuhan, Hubei, China; ^3^ Department of Rheumatology and Clinical Immunology, Peking Union Medical College Hospital, Chinese Academy of Medical Sciences and Peking Union Medical College, Beijing, China; ^4^ Department of Rheumatology and Department of Clinical Laboratory, Peking Union Medical College Hospital, Chinese Academy of Medical Sciences and Peking Union Medical College, Beijing, China; ^5^ Department of Physiology and Cell Biology, Wexner Medical Center, The Ohio State University, Columbus, OH, United States

**Keywords:** irritable bowel syndrome, biomarkers, autoantibodies, autoimmunity, HuProtTM microarrays

## Abstract

Immune activation and several autoantibodies might be involved in the pathophysiology of irritable bowel syndrome (IBS). We aimed to identify serum biomarkers for IBS by HuProt™ microarray. IBS patients met Rome III criteria were enrolled. Control groups included healthy controls (HCs) and disease controls (DCs). In stage I, we profiled sera from IBS and control groups with HuProt™ microarrays. Based on significant different proteins in stage I, IBS focused microarrays were constructed and validated in a larger cohort in stage II, then decision tree models were generated to establish a combination of biomarkers. In stage III, 4 purified proteins were verified by ELISA. Finally, we analyzed the correlation of autoantibodies with symptoms. In stage I, we identified 47 significant different proteins including 8 autoantibodies of IgG, 2 of IgA between IBS and HCs; 13 autoantibodies of IgG, 13 of IgA between IBS and DCs. In stage II, we found the positive rates of 14 IgG and IgA autoantibodies in IBS were significantly higher than HCs. Five autoantibodies of IgG and 7 IgA were comprehensively involved in differentiating IBS and HCs with the sensitivity and specificity to diagnose IBS as 40%–46.7% and 79.4%–86.3%. The median optical density value of ELAVL4 (IgG) and PIGP (IgA) were significantly higher in IBS than HCs. Parts of autoantibodies above were related to IBS symptoms. We found a combination of autoantibodies to differentiate IBS with HCs, but no specific autoantibodies could serve as serum biomarkers for IBS.

## Introduction

Irritable bowel syndrome (IBS) is a common functional bowel disorder characterized by abdominal pain or discomfort associated with altered bowel habits according to Rome III criteria. In the United States, IBS accounted for up to 12% of primary care visits and 28% of gastroenterology referrals (Everhart and Ruhl, 2009) and cost an estimated $20 billion annually, which severely compromised patients’ quality of life (Zhu et al., 2015). In China, the total direct medical costs estimated per patient per year for IBS patients in the whole disease course were USD 691.8 ± 1,067.2 (Fan et al., 2017). However, the pathophysiology of IBS is not well understood due to numerous factors playing multiple roles in disease development, such as diet (Böhn et al., 2015), stress, post-infectious changes, low-grade mucosal inflammation and disturbances in the intestinal microbiota (Pittayanon et al., 2019; Chey et al., 2020).

Nowadays, the diagnosis of IBS depended on symptom-based Rome criteria. However, data showed the sensitivity and specificity of the Rome III criteria to diagnose IBS were 69.6% and 82% respectively (Furman and Cash, 2011) and it was reported 70% of inflammatory bowel disease (IBD) patients fulfilled the IBS diagnostic criteria. (Sood et al., 2016) Moreover IBS was considered as a diagnosis of exclusion by 75% of community gastroenterologists and 23% of IBS experts. (Spiegel et al., 2010) In addition, 61% of IBS experts do not feel comfortable enough to confidently diagnose a patient as IBS solely based on criteria (Spiegel et al., 2010) thus many patients were investigated extensively with invasive radiographic and endoscopic imaging to make a diagnosis of exclusion. (Tibble et al., 2002) Chinese data showed IBS patients presented high rates of frequent healthcare-seeking and colonoscopies but low satisfaction rate to therapy (Fan et al., 2017).

IBS overlaps with other gastrointestinal diseases like IBD (Halpin and Ford, 2012) and celiac disease (Sainsbury et al., 2013) thus discovering a sensitive and specific biomarker is necessary to IBS diagnosis. Intensive research has been performed in this field and found potential biomarkers including systemic inflammation biomarkers interleukin-6 (IL-6), IL-8, macrophage inflammatory protein-1β (MIP-1β) (Pike et al., 2015); stool-based biomarkers of mucosal inflammation like calprotectin (Waugh et al., 2013); visceral hypersensitivity (Mertz et al., 1995); increased immune factors including lymphocytes, mast cells. (Cremon et al., 2009) Many of the above biomarkers were not IBS specific and their diagnostic utility and availability were low to moderate. (Camilleri et al., 2017).

Growing evidences showed immune activation was involved in the pathophysiology of IBS. Törnblom et al. found inflammation and enteric neuropathy in full-thickness biopsy tissues in IBS patients. (Törnblom et al., 2002) In addition, several autoantibodies were reported in sera of patients with IBS especially anti-enteric neuronal antibodies which could induce neuronal apoptosis *in vitro*. (De Giorgio et al., 2003; Wood et al., 2012; Li et al., 2016; Fan et al., 2018) Despite the enormous technological advances and the intense research performed, we still do not have any comprehensive studies that focused on the functions of antibodies or sera biomarkers in the pathophysiology of IBS. Proteins are the ultimate effector molecules of cellular functions. (Auger et al., 2009) Currently, protein microarrays have become a powerful proteomics tool for biomarker discovery. (Chen et al., 2008; Hu et al., 2012; Hu et al., 2016) Within a protein microarray, thousands of individually purified proteins were immobilized in a highly parallel, high-throughput way. (Duarte and Blackburn, 2017) Wood et al. used human protein microarray containing 8,000 proteins and discovered 3 antigens that related to IBS (Wood et al., 2012). The HuProt™ human proteome protein microarrays containing >19,000 specific proteins are the largest protein microarray platforms available to date (Sjöberg et al., 2016; Li et al., 2021). The aim of this study was to identify serum biomarkers (autoantibodies) for IBS and IBS with specific symptoms using HuProt™ human proteome microarray approach.

## Materials and methods

### Study design

**FIGURE 1:**
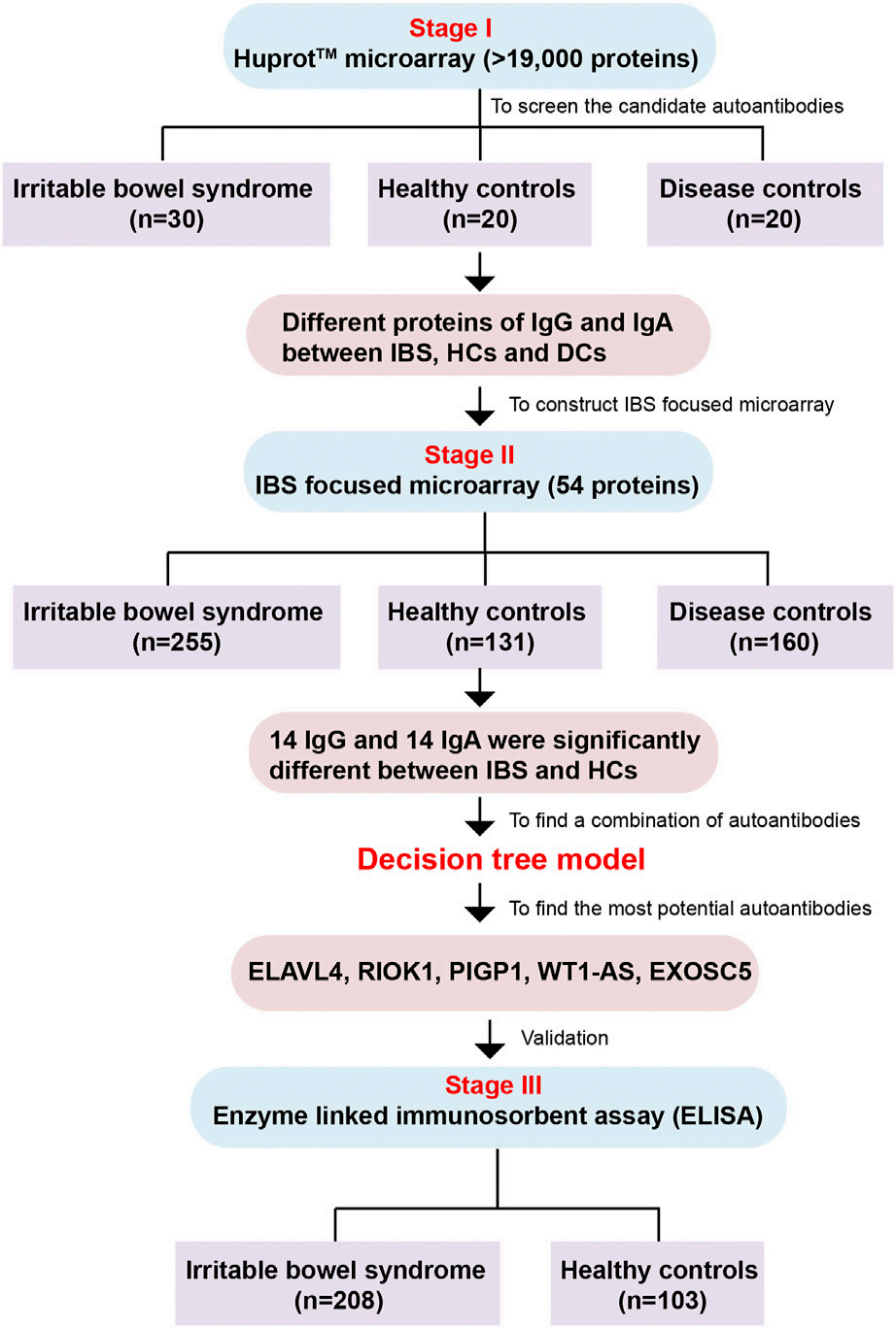
The flow chart of the whole study. The study is comprised of three stages including Huprot™ human proteome microarray screening, IBS focused microarray test and ELISA validation. IBS, irritable bowel syndrome; HCs, healthy controls; DCs, disease controls.

We designed a three-stage study to identify and validate the specific autoantibodies for IBS patients. Each serum sample was diluted and individually incubated on the HuProt™ microarrays, followed by a multiplexed detection of autoantigens that could be recognized by human sera proteins (autoantibodies) of the IgG and IgA isotypes. The protein spot on the microarrays will be positive when serum autoantibodies bind to corresponding autoantigens indicating the existence of autoantibodies in sera. In stage I, the HuProt™ version 3.0 human proteome microarray (Bernstein et al., 2010) was used to detect the possible different sera proteins between IBS patients (including different subtypes and disease severity), healthy controls (HCs) and disease controls (DCs) among a small cohort, which were called the candidate autoantigens associated with IBS in this study. Then the screened different autoantigens in stage I were constructed as the IBS focused microarray. In stage II, we used the IBS focused microarray to further identify the IBS-associated autoantibodies of sera in a much larger cohort, and then we used C4.5 algorithm through *Python* programming language to generate a decision tree model. In stage III, the most potential IBS-specific autoantigens validated in stage II were expressed and further validated *via* enzyme linked immunosorbent assay (ELISA) in a large cohort (Figure 1). Finally, we analyzed the correlation of IBS-associated autoantibodies with clinical symptoms.

### Subjects

Patients with IBS: Consecutive patients with IBS met Rome III diagnostic and subtype criteria were enrolled in the out-patient clinic of gastroenterology, Peking Union Medical College Hospital (PUMCH). Patients with organic gastrointestinal diseases, connective tissue diseases and metabolic diseases were excluded based on the results of routine tests for blood, urine, stool; liver, kidney, and thyroid function; measurements of carcinoembryonic antigen, erythrocyte sedimentation rate and C-reactive protein; and abdominal ultrasound and colonoscopy in the past year. Patients with pregnancy or lactation were excluded. Patients participated the study after being informed.

DCs: DCs groups included patients with slow transit functional constipation (STFC), chronic intestinal pseudo-obstruction (CIPO) and IBD. STFC patients fulfilled the Rome III diagnostic criteria of functional constipation and organic diseases were excluded. Gastrointestinal transit time (GITT) and anorectal manometry (ARM) supported they were having slow transit. Patients with IBD including ulcerative colitis (UC) and Crohn’s disease (CD) met the diagnostic criteria of World Gastroenterology Organization. (Downes et al., 2018).

HCs without symptoms were from the health examination center of PUMCH who had normal examination results. Sera of HCs were the remaining samples after routine tests.

All subjects were aged 18–65 years. The subjects of IBS, STFC and CIPO were enrolled from June 2009 to August 2017, sera of IBD and HCs were obtained from May 2014 to August 2017. The study was approved by the Ethics Committee of PUMCH (S234).

### IBS questionnaires

IBS patients completed symptom questionnaires including demographic data, symptom frequency and severity. Abdominal pain/discomfort was scaled as mild, moderate and severe according to patients’ report. Symptom score for IBS with diarrhea (IBS-D) was calculated according to Zhu, et al. (Zhu et al., 2015), with a total possible score of 15. We defined mild IBS as a symptom score ≤8, moderate IBS as 9–10, and severe IBS as >10 for patients with IBS-D based on symptom score percentiles (Fan et al., 2017). For patients with mixed IBS (IBS-M) and IBS with constipation (IBS-C), the symptom severity was determined by the severity and frequency of abdominal pain, number of other symptoms, health-related quality of life, and healthcare use. (Drossman, 2016) Persistent symptoms referred to having IBS symptom onset every day.

The Hamilton Anxiety (HAMA) and Hamilton Depression (HAMD) scales were used to evaluate patients’ psychological status by specially trained professionals through conversation and observation, the coexisting anxiety or depression were judged according to the instructions. (Hamilton, 1960).

### Serum sample collection

Serum samples were collected, separated and stored at −80°C until use.

### HuProt™ human proteome microarrays

The HuProt™ version 3.0 human proteome microarray (CDI Laboratories, Inc., Baltimore, Maryland, United States) contains >19,000 unique proteins (autoantigens). Recombinant proteins are expressed in the yeast *S. cerevisiae*, purified, and printed on glass slides that are coated with an ultra-thin layer of nitrocellulose film in duplicate. These expressed recombinant proteins are N-terminal Glutathione S-transferase (GST) and RGS-His6-tagged, and the quality of each microarray batch is determined by GST immunoblotting (98% of all proteins show GST signals significantly higher than negative controls) (Supplementary Figure S1A, 1B). The correlation coefficient between duplicate spots for each protein was 0.945 ensuring high reproducibility (Supplementary Figure S1C). Positive controls (histone1 [H1], H2A + B, H3, H4) and negative controls (bull serum albumin [BSA] and buffer) were spotted in duplicates on the microarrays to ensure the integrity of the experiments at various steps. Supplementary Figure S1D showed the quality control of IBS focused microarrays determined by GST immunoblotting.

### HuProt™ human proteome microarrays for screening the candidate autoantigens associated with IBS - stage I

The HuProt™ human proteome microarrays were taken out from -80°C and equilibrated to room temperature (RT) for 20 min. Add 5 ml of blocking solution (5% BSA in phosphate buffered solution with 0.1% Tween 20 [PBST]) to each compartment in the 4-well plates and incubate the microarrays at RT for 1.5 h with gentle shaking (40 rpm). Serum samples diluted 1:1,000 fold into 5 ml blocking solution were added and incubated at RT with gentle shaking for 1 h. After 3 × 10 min washes with PBST, the microarray was incubated with 5 ml of 1:1,000 diluted Alexa 647 conjugated goat anti-Human IgG and Cy3-conjugated rabbit anti-Human IgA (Jackson Laboratory, Bar Harbor, ME, United States) at RT for 1 h with gentle shaking in dark. After 3 × 10 min washes with PBST and 3 × 10 min washes with double-distilled H_2_O, the microarray was dried by centrifuging at 2000 rpm for 1 min in a 50 ml centrifuge tube. Finally, the microarray was scanned with the GenePix 4000B Microarray Scanner (Molecular Devices, Sunnyvale, CA, United States) using optimal settings (power = 10, photomultiplier tube = 700) in a 635-nm channel and a 532-nm channel simultaneously and analyzed with GenePix Pro 6.0 software (Molecular Devices, Sunnyvale, CA, United States).

### Construction of IBS focused microarray for identifying the IBS-associated autoantigens - stage II

Through the data of HuProt™ human proteome microarray in stage I, together with 7 previously reported autoantigens related to gut motility disorders and enteric neuropathy, including ELAVL3, ELAVL4, SNRPA, DUSP11, PNMA2, CACNG3 and GAD1 (Törnblom et al., 2007), fifty-four candidate autoantigens potentially associated with IBS were purified and printed in duplicate in 14 identical sub-arrays on SuperEpoxy2 (glass) Slide™ to construct the IBS focused microarrays (CDI Laboratories, Inc., Baltimore, Maryland, United States). A 14-hole rubber gasket was applied to form 14 individual chambers.

The procedures in stage II were similar to that of the stage I of HuProt™ human proteome microarray. While in the serum assay process, 50 μl of 1:1,000 diluted human serum was sequentially incubated in each chamber. Then the rubber gaskets were removed carefully, and the microarrays were washed and scanned.

### Microarray data analysis

The scanned images in stage I and II were analyzed by GenePix Pro software to get raw data files (gpr files). The GenePix array list (.gal file) is aligned and resized to fit the individual spot features that can describe the location of each protein on the array. For HuProt™ human proteome microarray assays, the median foreground and background intensity for each spot were acquired. The ratio of foreground to background signals for each spot was regarded as the spot’s signal intensity, the mean signal value of each duplicate pair was the final protein’s signal intensity. Signal intensities were normalized and the cut-off was calculated according to procedures described previously by Hu et al. (Duarte and Blackburn, 2017) We firstly identified proteins with normalized signal intensities less than 1. Symmetric pseudo-data for the right side of the axis (x = 1) were generated to estimate the standard deviation (SD). The mean of the normalized signal intensities for all spots in a microarray was determined. We set the cut-off as mean +5 SD of the signal intensity of all the proteins in a HuProt™ human proteome microarray of HCs and DCs and the spot with signal intensity greater than the cut-off was identified as ‘positive’ hit. Then we calculated the mean signal intensity of each protein in IBS, HCs and DCs. The ratio of mean signal intensity of each protein in IBS group to HCs or DCs is called fold change value. *t*-test was chosen to assess the differential significance of each protein between IBS and HCs or DCs based on signal intensity. Besides, we also took IBS subgroup difference into account and compared the mean signal intensity between mild-moderate and severe IBS, and calculated the corresponding fold change value.

In order to identify the possible autoantibodies maximally and cost-effectively, we set to find about fifty candidate autoantigens potentially associated with IBS during the stage I and construct as the IBS focused microarray. We set IBS-associated candidate autoantigens IgG as *p* value of *t*-test less than 0.05 and fold change >1.9, while that of IgA as *p* value less than 0.05 and fold change >2.3. Also, we added significant different autoantigens between mild-moderate and severe IBS by IgG as *p* value of *t*-test less than 0.05 and fold change >1.9, and that of IgA as *p* value less than 0.05 and fold change >2.3.

For the focused microarrays fabricated with potential IBS-associated autoantigens, the signal intensity for each protein was defined by ratio (dividing foreground intensity by background intensity). According to data of HCs, we set the signal intensity of mean +2 SD of HCs as the cut-off to identify the positives. Through comparing IBS and control groups using chi-square test, the proteins with *p* value <0.05 were considered as statistically significant IBS-associated autoantigens.

### Decision tree model for selection of combination of autoantigens

Then we used C4.5 algorithm through *Python* programming language (Reddy and Chittineni, 2021) to generate a decision tree model. The statistically significant IBS-associated autoantigens were put into the model. At each node of the tree, C4.5 chooses the attribute of the data that most effectively splits its set of samples into subsets enriched in one class or the other. The splitting criterion is the normalized information gain. The attribute with the highest normalized information gain is chosen to make the decision.

### ELISA validation for the most promising IBS-associated autoantigens -stage III

We selected 5 most potential IBS-associated autoantigens according to the results of decision trees and previous reports, including two most important proteins on the top of decision trees RIOK1 (IgG, also present in IgA decision tree) and PIGP (IgA), and EXOSC5 (present in IgG and IgA decision tree), WT1-AS, ELAVL4 to validate in stage III. Finally, the purified recombinant proteins (CDI Laboratories Inc., Baltimore, Maryland, United States) including WT1-AS, ELAVL4, EXOSC5, PIGP were further verified by ELISA (RIOK1 was not successfully purified). A total of 100 μl verified recombinant proteins were coated onto 96-well plates at 4°C overnight with a concentration of 75 ng/100 μl (WT1-AS, EXOSC5, PIGP), or 200 ng/100 μlL (ELAVL4). Nonspecific binding was blocked with 200 μl PBST containing 3% BSA/well at 4°C overnight. The following day, the wells were incubated with human sera (1:100) including IBS and HCs randomly selected from the same cohort in stage II (age and gender matched) at 37°C for 1 h and then washed three times with 400 μl/well of PBST. Control group was incubated with PBS. Subsequently, 100 μl of horseradish peroxidase-labeled goat anti-human IgG monoclonal antibody for WT1-AS, ELAVL4 and EXOSC5 (1:1,000) and goat anti-human IgA monoclonal antibody (1:1,000) for PIGP were added to each well and incubated at 37°C for 1 h. After three washes with 400 μl/well of PBST, 100 μl of tetramethylbenzidine substrate solution was added and incubated for 90 s at RT. The reaction was terminated by addition of 100 μl of ELISA stop solution (Solarbio) and immunoreactivity was measured by reading the A450 (optical density value, OD value) (Tecan Sunrise, Männedorf, Switzerland).

### Statistical analysis

All analyses were performed using SPSS version 19.0 (IBM Corporation, Somers, NY, United States). Comparisons between the two groups were made by Student’s t-tests for parametric data and Mann–Whitney *U* test for nonparametric data. Chi-square tests were used for categorical variables. *p* < 0.05 was considered statistically significant.

## Results

### Demographic data

In stage I, 30 patients with IBS including IBS-D, IBS-C and IBS-M with mild, moderate and severe symptoms were enrolled respectively. Patients with STFC (*n* = 7), IBD (5 with UC and 1 with CD) and CIPO (*n* = 7) were included in DCs and 20 HCs were enrolled.

In stage II, a larger cohort consisted of 255 patients with IBS (including IBS-D, IBS-C and IBS-M with mild, moderate and severe abdominal pain/discomfort), 131 HCs and 160 DCs (including 89 patients with STFC, 47 patients with UC and 24 with CD) were enrolled. Among IBS cases, 101 patients had persistent symptom onset; 221 patients completed HAMA and HAMD evaluation, 59.6% and 26.8% patients were coexisted with anxiety and depression.

In stage III, 208 IBS and 103 HCs were randomly selected from the same cohort in stage II. Demographic data of each group were showed in Table 1.

**TABLE 1:** Demographic data of IBS, HCs and DCs in the three study stages.

**Variables**	**Stage I**			**Stage II**			**Stage III**	
	**IBS (*n* = 30)**	**HCs (*n* = 20)**	**DCs (*n* = 20)**	**IBS (*n* = 255)**	**HCs (*n* = 131)**	**DCs (*n* = 160)**	**IBS (*n* = 208)**	**HCs (*n* = 103)**
Age	47.1 ± 12.0	44.8 ± 11.1	39.0 ± 10.9	40.3 ± 11.4	37.1 ± 9.7	43.3 ± 14.0	40.4 ± 11.8	37.5 ± 9.3
M: F	14:16	8:12	10:10	164:91	50:81	68:92	136:72	38:65
Classification	IBS-D (24)		IBD (6)	IBS-D (240)		STFC (89)	IBS-D (195)	
	IBS-C (2)		STFC (7)	IBS-C (6)		IBD (71)	IBS-C (4)	
	IBS-M (4)		CIPO (7)	IBS-M (9)			IBS-M (9)	
	Mild IBS (6)			Mild IBS (65)				
	Moderate IBS (16)			Moderate IBS (126)				
	Severe IBS (8)			Severe IBS (64)				
				Mild pain/discomfort (50)				
				Moderate pain/discomfort (161)				
				Severe pain/discomfort (44)				
				Anxiety (97) *				
				Severe anxiety (62) *				
				Depression (103) *				
				Severe depression (6) *				

IBS, irritable bowel syndrome; HCs, healthy controls; DCs, disease controls; IBS-D, IBS, with diarrhea; IBS-C, IBS, with constipation; IBS-M, mixed IBS; IBD, inflammatory bowel disease; STFC, slow transit functional constipation; CIPO, chronic intestinal pseudo-obstruction; M, male; F, female. * A total of 221 patients finished HAMA, and HAMD, evaluation.

### Candidate autoantigens associated with IBS in stage I

In stage I, we identified 47 IBS-associated candidate autoantigens including 8 significant candidate autoantigens IgG, 2 IgA between IBS and HCs; 13 significant candidate autoantigens IgG, 13 IgA between IBS and DCs; 9 IgG and 2 IgA candidate autoantigens between IBS subgroups. Supplementary Table S1 listed the significant candidate autoantigens, the fold change value and *p* value. Supplementary Figure S2 presents 4 typical different proteins between IBS and HCs group or DCs group on HuProt™ microarrays.

### IBS-associated autoantigens in stage II

In stage II, we found that the positive rates of 14 IgG autoantigens (including RIOK1, ANXA1, ELAVL4, EXOSC5, *etc.*) and 14 IgA (including PIGP, CACNG3, RIOK1, VCY, EXOSC5, *etc.*) of IBS patients were significantly higher than HCs but had no significant difference with DCs (Table 2).

**TABLE 2:** Comparison of the positive rates of IBS-associated autoantigens identified by IBS focused microarrays (Stage II).

	Proteins	IBS (*n* = 255)	HCs (*n* = 131)	DCs (*n* = 160)	*P*-value^a^	*P*-value^b^
IgG	RIOK1	16.1	5.3	21.9	0.002*	0.137
	ANXA1	15.3	7.6	17.5	0.032*	0.552
	ELAVL4	14.5	6.1	20.6	0.015*	0.105
	EXOSC5	14.5	4.6	13.8	0.003*	0.829
	FAM46B	14.1	6.1	18.8	0.019*	0.209
	APBA1	13.7	4.6	16.2	0.006*	0.48
	WDR83	13.3	5.3	17.5	0.016*	0.246
	WT1-AS	12.9	5.3	16.2	0.020*	0.347
	PRKCDBP	12.5	3.8	20.0	0.006*	0.057
	DAB1	11.8	5.3	15.6	0.042*	0.259
	TRMT2A	11.4	4.6	16.9	0.028*	0.110
	SNRPA	9.8	3.8	13.1	0.037*	0.294
	TFAP2E	9.0	2.3	13.1	0.012*	0.186
	WDR54	9.0	2.3	6.9	0.012*	0.438
IgA	PIGP	16.9	4.6	18.1	0.001*	0.741
	CACNG3	12.5	4.6	12.5	0.013*	0.988
	RIOK1	12.5	4.6	11.2	0.013*	0.692
	VCY	12.2	4.6	10	0.017*	0.50
	EXOSC5	12.2	4.6	7.5	0.017*	0.130
	PAGE5	11.8	5.3	7.5	0.042*	0.161
	PPM1K	11.8	5.3	18.1	0.042*	0.071
	GPBP1_frag	11.4	4.6	9.4	0.028*	0.520
	HCLS1	11.4	3.8	12.5	0.013*	0.729
	ANXA1	11.0	3.8	11.9	0.017*	0.780
	WDR83	11.0	4.6	11.2	0.036*	0.932
	ZNF667	10.2	3.8	13.1	0.029*	0.359
	TCEAL6	9.8	3.8	16.9	0.037*	0.052
	PTRH2	9.8	3.1	7.5	0.017*	0.423

Data presented as percentage of patients or subjects. IBS, irritable bowel syndrome; HCs, healthy controls; DCs, disease controls. P-value^a^, *p* value for IBS, vs. HCs; P-value^b^, *p* value for IBS, vs. DCs. *p < 0.05.

Overall, the positive rate of containing any IgG autoantibody among the 14 IgG autoantibodies is significantly higher in IBS than HCs (46.7% vs. 27.5%, *p* < 0.001), the positive rate of containing any IgA autoantibody among the 14 IgA autoantibodies is significantly higher in IBS than HCs (55.7% vs. 32.8%, *p* < 0.0001), and the positive rate of containing any IgG/IgA autoantibody is significantly higher in IBS than HCs (69.0% vs. 42.7%, *p* = 0.019).

Table 3 showed the significant differences of IBS-associated autoantigens IgG and IgA between IBS patients with severe symptoms and HCs, IBS patients with persistent symptoms and HCs. In addition, the positive rates of WT1-AS, RIOK1 IgG (18.2% vs. 5.3%, *p* = 0.008; 15.9% vs. 5.3%, *p* = 0.025) and PIGP, TCEAL6, GPBP1_frag IgA (20.5% vs. 5.3%, *p* = 0.001; 18.2% vs. 3.8%, *p* = 0.002; 15.9% vs. 5.3%, *p* = 0.013) were significantly higher in IBS patients with severe abdominal pain/discomfort than HCs. The positive rates of RIOK1, WDR83, DTNBP1, WDR54 IgG (23.4% vs. 5.3%, *p* = 0.014; 14.5% vs. 5.3%, *p* = 0.031; 14.5% vs. 4.6%, *p* = 0.016; 12.9% vs. 2.3%, *p* = 0.003) and RIOK1, CACNG3, GAD1 IgA (17.7% vs. 4.6%, *p* = 0.003; 14.5% vs. 4.6%, *p* = 0.016; 11.3% vs. 2.3%, *p* = 0.008) were significantly higher in IBS patients with severe anxiety than HCs. Since the number of IBS patients with severe depression was only 6, we did not analyze the difference between IBS patients with severe depression and HCs.

**TABLE 3:** Comparison of the positive rates of IBS-associated autoantigens among IBS subgroups and HCs.

	Proteins	Severe IBS (%)	HCs	*p*-value	Proteins	Persistent IBS (%)	HCs	*p*-value
**IgG**	RIOK1	23.4	5.3	0.001*	RIOK1	17.8	5.3	0.002*
	ANXA1	20.3	7.6	0.01*	ANXA1	15.8	7.6	0.016*
	FAM46B	20.3	6.1	0.003*	APBA1	15.8	4.6	0.004*
	ELAVL4	18.8	6.1	0.006*	FAM46B	15.8	6.1	0.016*
	EXOSC5	18.8	4.6	0.001*	ELAVL4	14.9	6.1	0.027*
	APBA1	17.2	4.6	0.003*	EXOSC5	14.9	4.6	0.007*
	WDR83	15.6	5.3	0.017*	WDR83	12.9	5.3	0.043*
	DAB1	15.6	5.3	0.017*	DAB1	12.9	5.3	0.043*
	ELAVL3	15.6	5.3	0.017*	SNRPA	11.9	3.8	0.019*
	WT1-AS	15.6	5.3	0.017*	PRKCDBP	10.9	3.8	0.035*
	SNRPA	15.6	3.8	0.004*				
	PPM1K	14.1	5.3	0.037*				
	PCDHGA10	14.1	4.6	0.02*				
	TRMT2A	14.1	4.6	0.02*				
**IgA**	PIGP	17.2	4.6	0.003*	RIOK1	17.8	4.6	0.001*
	ANXA1	14.1	3.8	0.016*	PIGP	15.8	4.6	0.004*
	EXOSC5	14.1	4.6	0.041*	GPBP1_frag	14.9	4.6	0.007*
	GPBP1_frag	14.1	4.6	0.041*	EXOSC5	12.9	4.6	0.022*
	PPM1K	14.1	5.3	0.037*	HCLS1	11.9	3.8	0.019*
	ZNF667	12.5	3.8	0.032*	WDR83	11.9	4.6	0.039*

Severe IBS, irritable bowel syndrome patients with severe symptoms; HCs, healthy controls; Persistent IBS, irritable bowel syndrome patients with persistent symptoms. *p < 0.05.

The detailed information of the significantly different autoantigens was showed in Supplementary Table S2, which were summarized from Uniprot Knowledgebase (http://www.uniprot.org).

### Diagnostic power of combination of autoantigens in decision tree model

**FIGURE 2:**
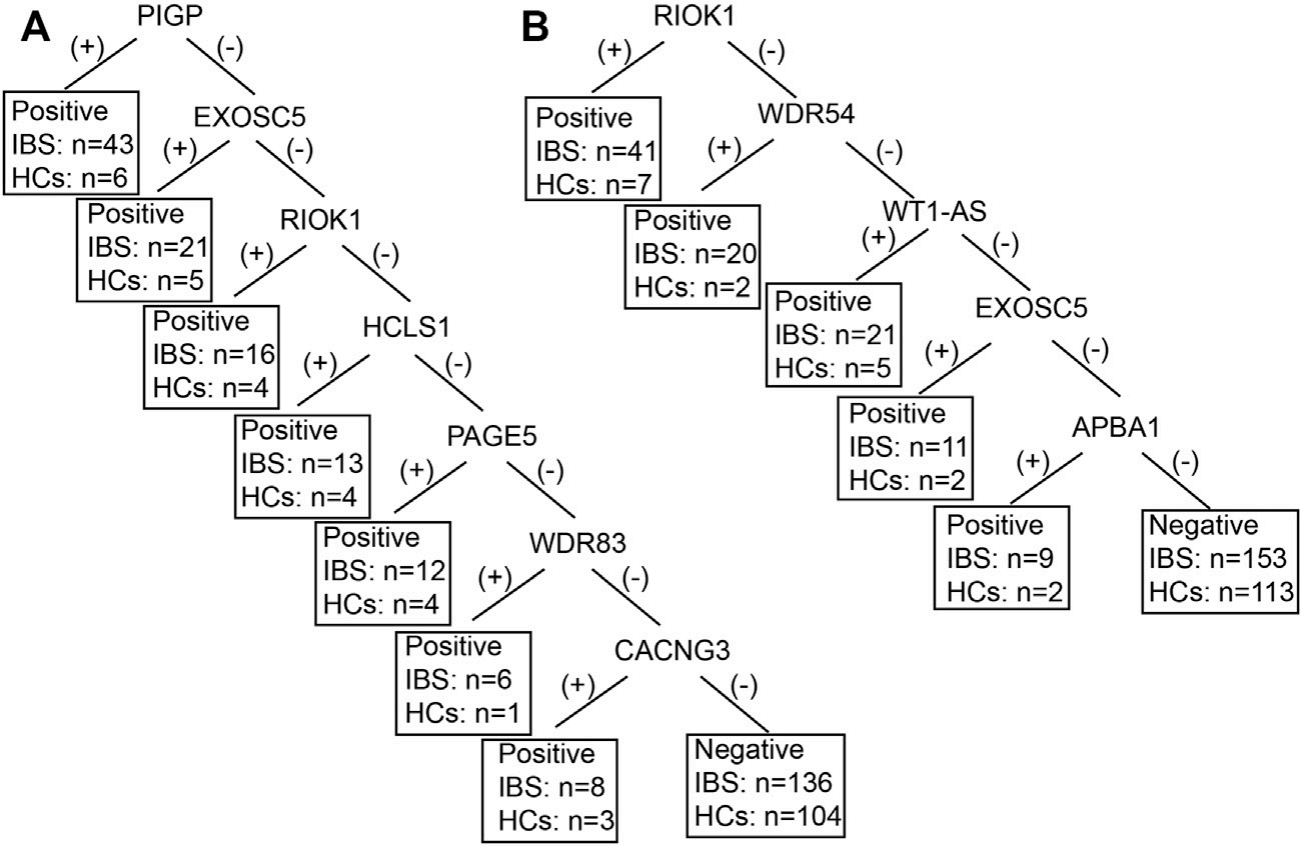
The decision tree model of IBS-associated autoantigens IgA **(A)** and IgG **(B)** between IBS patients and HCs. IBS, irritable bowel syndrome; HCs, healthy controls. For example, in figure B, if the RIOK was (+), IBS can be diagnosed, 41 IBS were diagnosed as IBS, 7 HCs were diagnosed as IBS. If the RIOK was (−), then WDR54 was tested. If the WDR54 was (+), IBS can be diagnosed, 20 IBS were diagnosed as IBS, 2 HCs were diagnosed as IBS and so forth. Positive refers to IBS can be diagnosed. Negative refers to healthy control can be diagnosed.

We only included IBS patients and HCs in the decision tree model since the positive rates of the autoantibodies were not significantly different between IBS patients and DCs. Figure 2 showed the decision tree model of IgA (A) and IgG (B) between the two groups. Five autoantigens IgG and 7 autoantigens IgA were comprehensively involved in differentiating IBS patients and HCs. The sensitivity and specificity of the decision tree model to diagnose IBS were 40% (102/255, IgG), 86.26% (113/131, IgG), and 46.67% (119/255, IgA), 79.39% (104/131, IgA) respectively.

**FIGURE 3:**
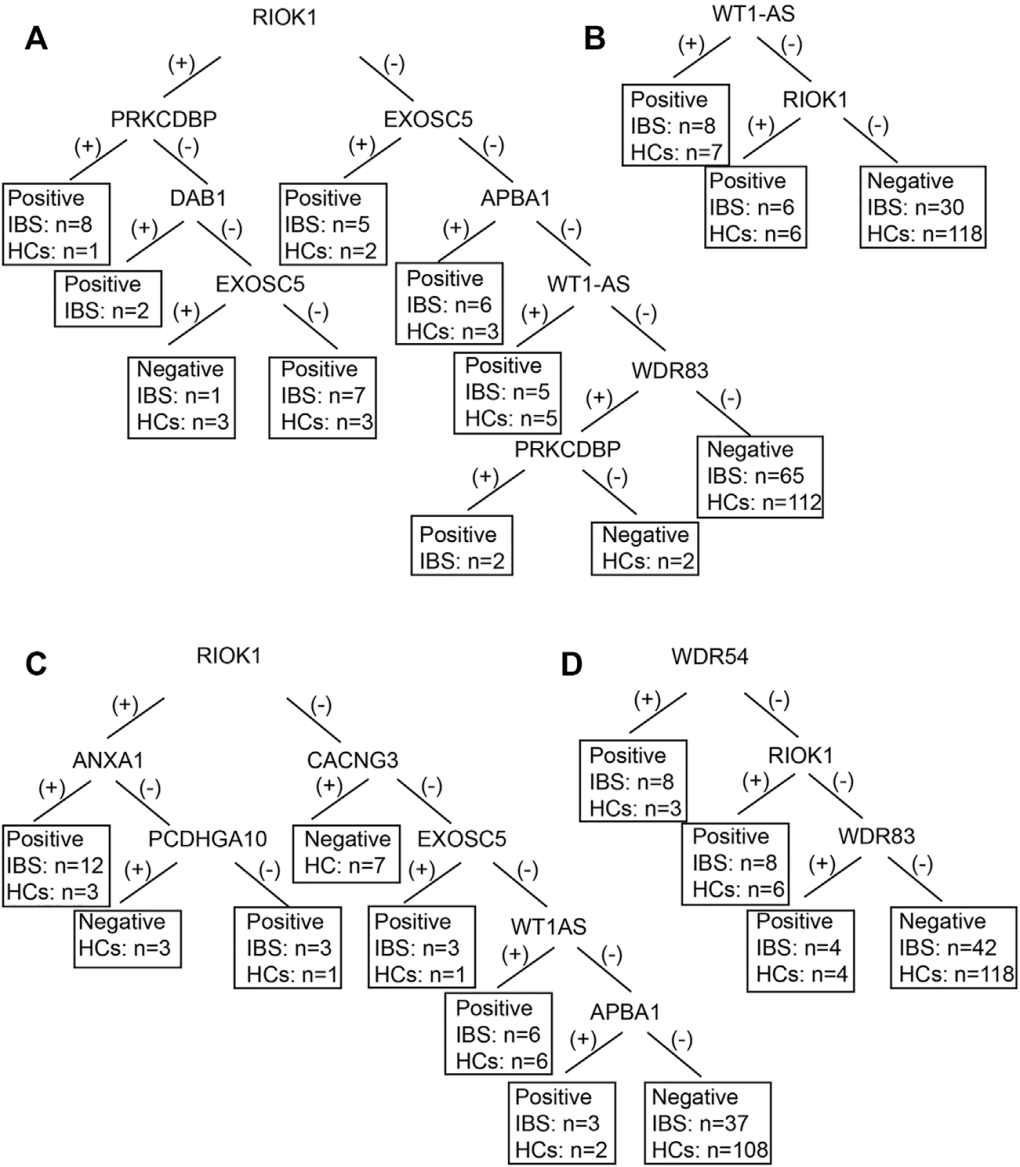
The decision tree models of IgG between IBS subgroup patients and HCs. Negative refers to healthy control can be diagnosed. **(A)** IBS patients with persistent symptoms and HCs. Positive refers to IBS with persistent symptoms can be diagnosed. **(B)** IBS patients with severe abdominal pain/discomfort and HCs. Positive refers to IBS with severe abdominal pain/discomfort can be diagnosed. **(C)** IBS patients with severe symptoms and HCs. Positive refers to IBS with severe symptoms can be diagnosed. **(D)** IBS patients with severe anxiety and HCs. Positive refers to IBS with severe anxiety can be diagnosed.

**FIGURE 4:**
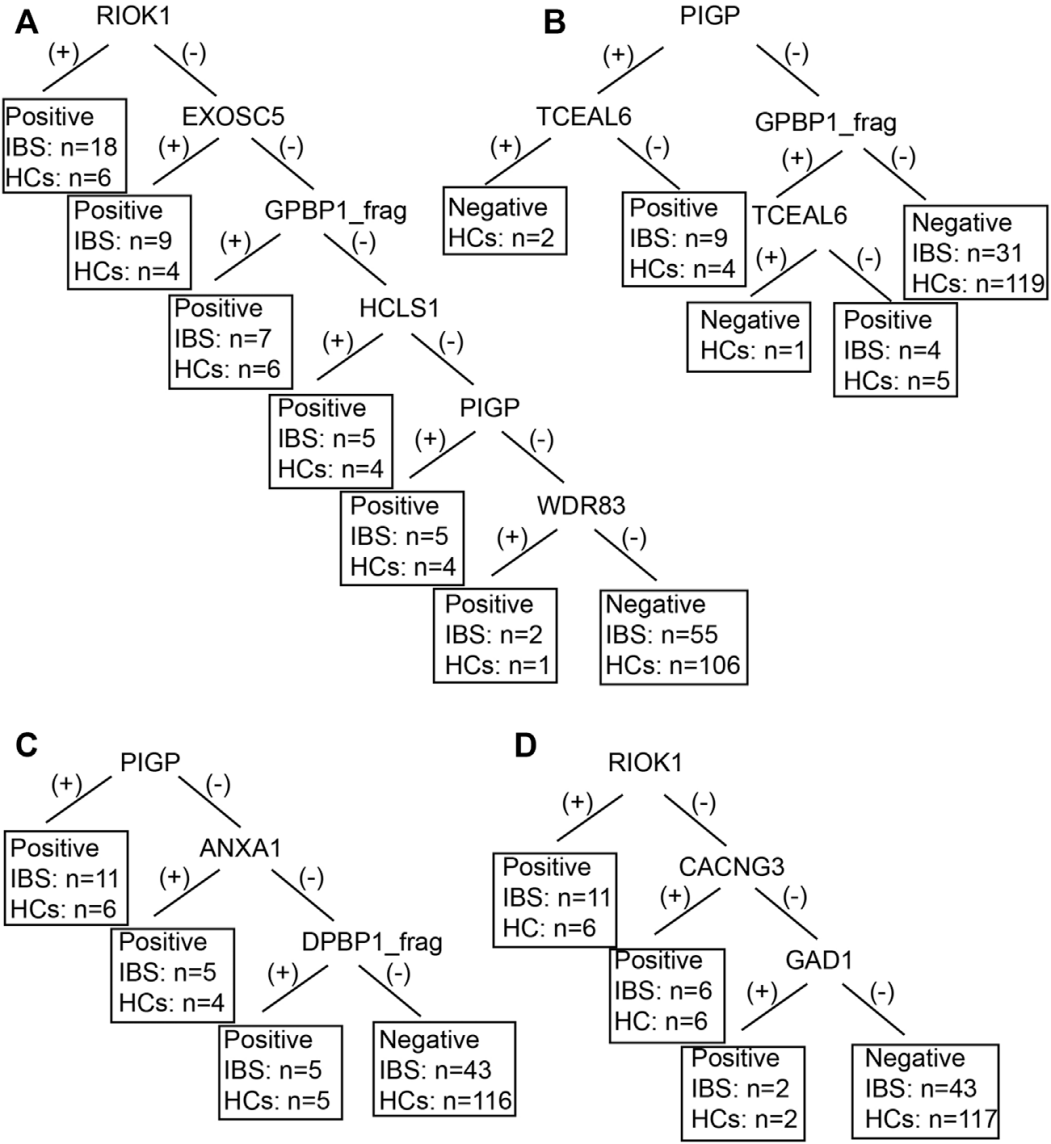
The decision tree models of IgA between IBS subgroup patients and HCs. Negative refers to healthy control can be diagnosed. **(A)** IBS patients with persistent symptoms and HCs. Positive refers to IBS with persistent symptoms can be diagnosed. **(B)** IBS patients with severe abdominal pain/discomfort and HCs. Positive refers to IBS with severe abdominal pain/discomfort can be diagnosed. **(C)** IBS patients with severe symptoms and HCs. Positive refers to IBS with severe symptoms can be diagnosed. **(D)** IBS patients with severe anxiety and HCs. Positive refers to IBS with severe anxiety can be diagnosed.

We also established subgroup decision tree models to identify subgroup specific autoantigens. Figures 3, 4 showed the decision tree models of IgG and IgA between IBS patients with persistent symptoms and HCs (Figures 3A, 4A), IBS patients with severe abdominal pain/discomfort and HCs (Figures 3B, 4B), IBS patients with severe symptoms and HCs (Figures 3C, 4C), IBS patients with severe anxiety and HCs (Figures 3D, 4D). In IgG, there were 7, 2, 7 and 3 autoantigens involved to differentiate IBS subgroups and HCs. The sensitivity and specificity of the decision tree model of IgG to differentiate IBS subgroups and HCs were 34.65% (35/101) and 89.31% (117/131) for IBS patients with persistent symptoms, 31.82% (14/44) and 90.08% (118/131) for IBS patients with severe abdominal pain/discomfort, 42.19% (27/64) and 90.08% (118/131) for IBS patients with severe symptoms, 32.26% (20/62) and 90.08% (118/131) for IBS patients with severe anxiety. In IgA, there were 6, 3, 3 and 3 autoantigens involved to differentiate IBS subgroups and HCs. The sensitivity and specificity of the decision tree model of IgA to differentiate IBS subgroups and HCs were 45.54% (46/101) and 80.92% (106/131) for IBS patients with persistent symptoms, 29.55% (13/44) and 93.13% (122/131) for IBS patients with severe abdominal pain/discomfort, 32.81% (21/64) and 88.55% (116/131) for IBS patients with severe symptoms, 30.65% (19/62) and 89.31% (117/131) for IBS patients with severe anxiety.

### The most promising IBS-associated autoantigens in stage III

**FIGURE 5:**
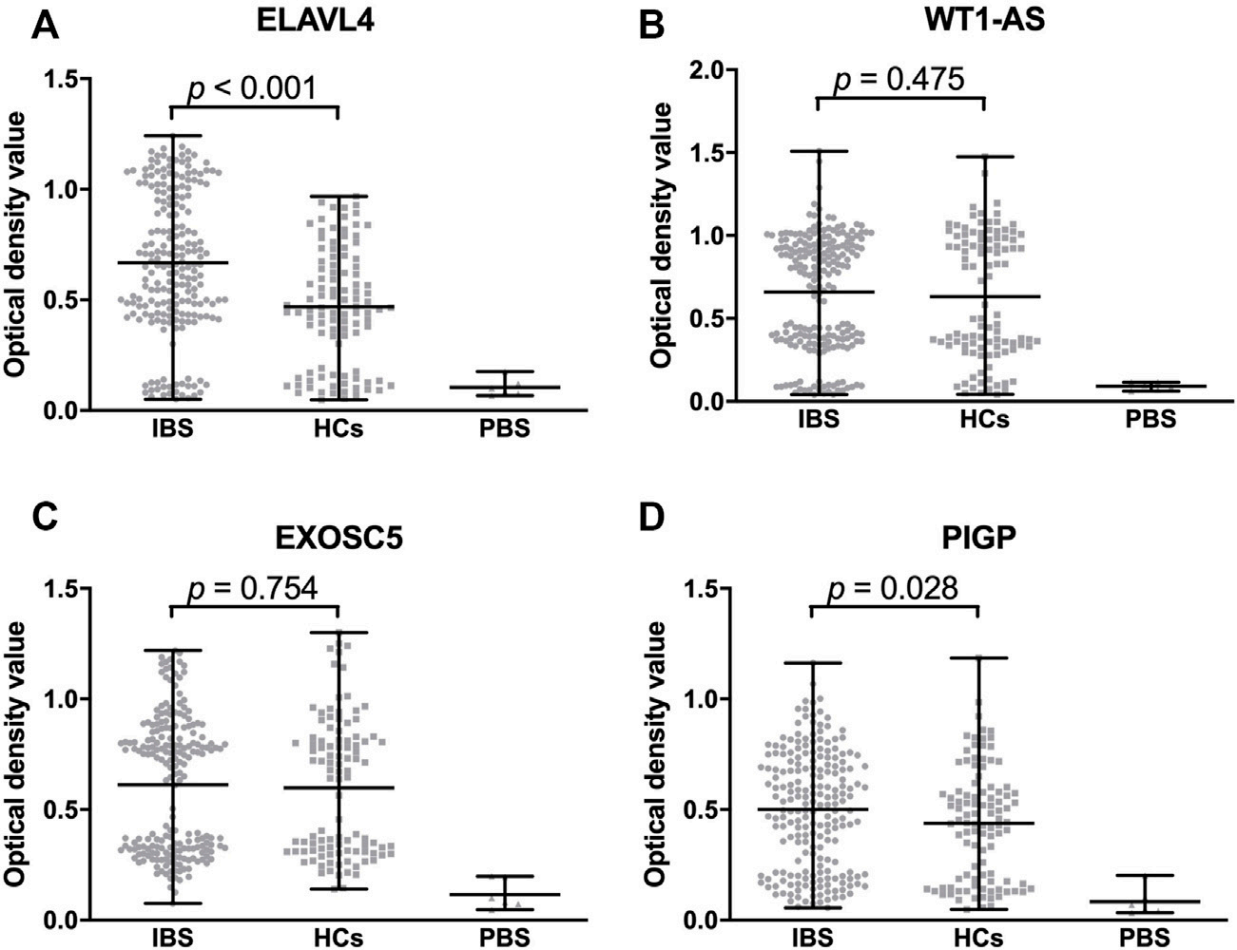
Comparison of optical density value of validated proteins among IBS patients, HCs and DCs. The optical density value of ELAVL4 (IgG) of IBS is significantly higher than HCs **(A)**. The optical density values of WT1-AS and EXOSC5 (IgG) are not significantly different between IBS and HCs **(B,C)**. The optical density value of PIGP (IgA) of IBS is significantly higher than HCs **(D)**. IBS, irritable bowel syndrome; HCs, healthy controls; PBS, phosphate buffered solution.

**FIGURE 6:**
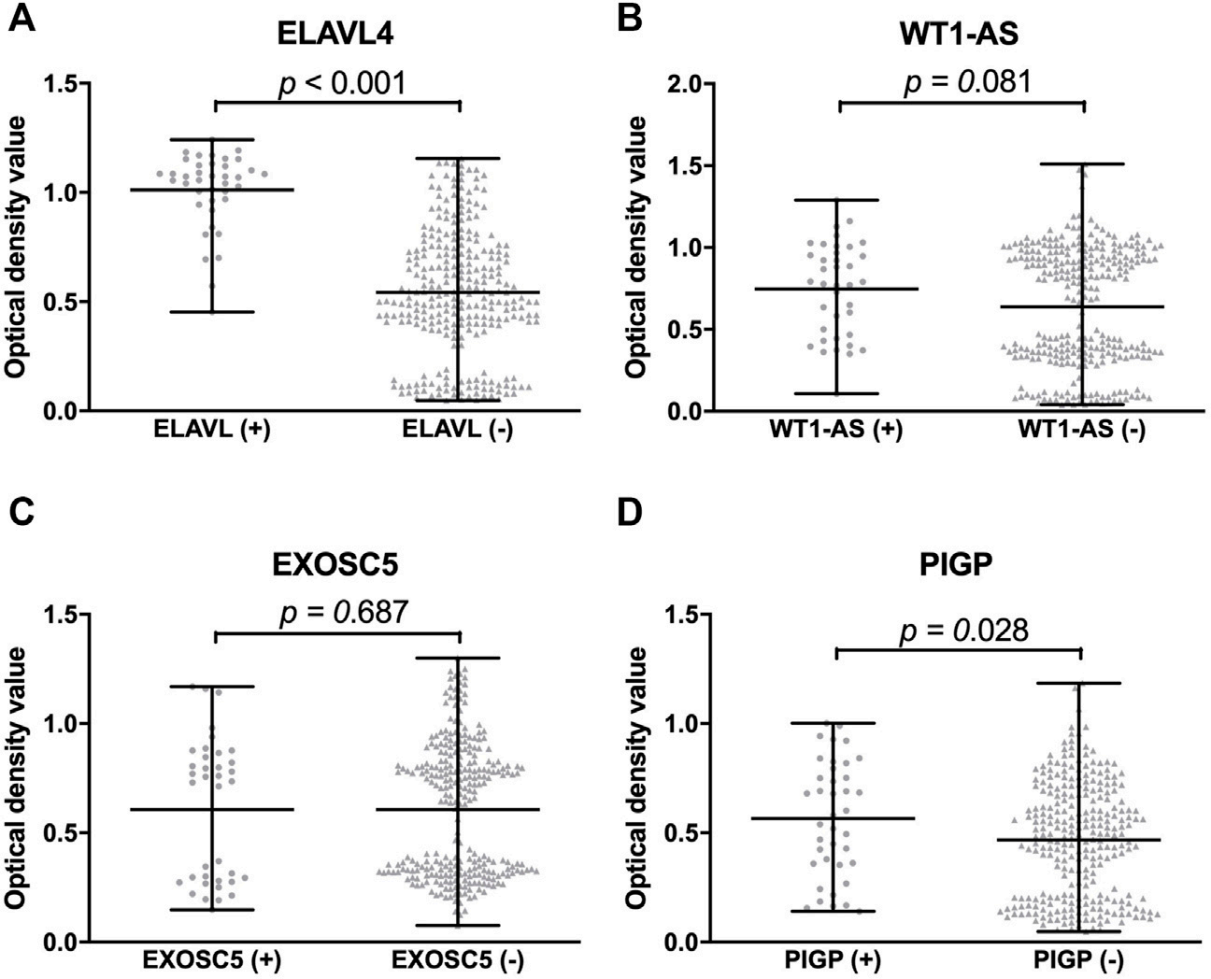
Comparison of optical density value of validated proteins between protein positive group and negative group by IBS focused microarray. The optical density value of ELAVL4 (IgG) of protein positive group is significantly higher than negative group by IBS focused microarray **(A)**. The optical density values of WT1-AS and EXOSC5 (IgG) are not significantly different between protein positive group and negative group **(B,C)**. The optical density value of PIGP (IgA) of protein positive group is significantly higher than negative group by IBS focused microarray **(D)**.

In the ELISA validation, the median OD value of ELAVL4 IgG and PIGP IgA was significantly higher in IBS than HCs (Figure 5). While no significant difference of the OD value of WT1-AS IgG, EXOSC5 IgG was observed between the two groups (Figure 5). Moreover, the median OD value of ELAVL4 IgG and PIGP IgA was significantly higher in ELAVL, PIGP positive subjects according to IBS focused microarray results than negative subjects (Figure 6). The median OD value of WT1-AS IgG, EXOSC5 IgG was not significantly different between WT1-AS, EXOSC5 positive subjects and negative subjects according to IBS focused microarray results (Figure 6).

### The correlation of IBS-associated autoantibodies with clinical symptoms

IBS patients who contain >3 significant IgG autoantibodies have a longer disease course (years) than IBS patients who contain ≤3 significant IgG autoantibodies (11.6 ± 9.9 vs. 6.7 ± 6.3, *p* = 0.004). The percentage of watery stools in IBS patients who contain >3 significant IgA autoantibodies was significantly higher than IBS patients who contain ≤3 significant IgA autoantibodies (27.3% vs. 11.1%, *p* = 0.028).

We compared the difference of clinical data between each significant autoantibody positive IBS patients and negative patients. The disease course (year) of anti-ELAV4 IgG positive IBS patients was longer than negative patients (12.0 ± 10.5 vs. 7.7 ± 6.5, *p* = 0.046). Anti-PIGP IgA and anti-TCEAL6 IgA positive IBS patients had more days of defecated-associated abdominal pain/discomfort per month than negative patients (23.0 ± 8.0 vs. 20.0 ± 8.0, *p* = 0.041; 25.0 ± 6.0 vs. 21.0 ± 8.0, *p* = 0.037). Moreover, the IBS symptom score of anti-RIOK IgA positive IBS patients was higher than negative patients (10.5 ± 1.9 vs. 9.4 ± 1.6, *p* = 0.004).

## Discussion

In the current study, we applied the HuProt™ human proteome microarray to systemically detect serum autoantibodies (target to autoantigens) in patients with IBS compared to HCs and DCs. By the three-stage study, we demonstrated that the positive rates of 14 IgG autoantibodies and 14 IgA autoantibodies in sera of IBS patients were significantly higher than HCs but had no significant difference with DCs. We also found the associations of autoantibodies with IBS symptoms, severity and disease course. Different from classical autoimmune diseases (Sjöberg et al., 2016), a diversity of autoantibodies rather than specific ones was detected in IBS, and the positive rates were relatively low.

The diagnosis of IBS is based on the presence of relatively specific symptoms grouped as clinical criteria, and in the exclusion of other gastrointestinal diseases with similar symptoms. The lack of a specific and sensitive biomarker for IBS has led to development of many biomarker discovery approaches in recent years. In addition to systemic inflammation biomarkers and intestinal mucosal expression of RNA about inflammatory cytokines (Aerssens et al., 2008; Swan et al., 2013), many studies focused on differently expressed proteins in serum samples between IBS patients and healthy controls by mass spectrometry. (Tsigaridas et al., 2018; Weaver et al., 2018) Previous studies identified 8 differently expressed proteins (IGKC, LAC3, APOE, CLUS, TRFE, APOH, XIV and COEA1) between IBS patients (*n* = 30) and healthy individuals (*n* = 10). (Tsigaridas et al., 2018) Another study by Kristen et al. found TGFβ1, PF4V1, PF4, APP, MMP9, PPBP, CTGF, SRGN, THBS1, WRN, LTBP1 (Isoform 3), and IGLV5-48 were significantly different in levels of expression of serum samples between IBS-C patients and healthy controls (*n* = 5 in each group). (Weaver et al., 2018) However, these results were obtained from smaller samples and the method of mass spectrometry covered limited proteins.

The HuProt™ version 3.0 human proteome microarray used in this study contained the entire known proteins of humans and we also verified the screened proteins in a much larger cohort. Protein microarray is a powerful and feasible tool for studying systematically protein-protein interactions and host-pathogen interactions, which has been widely used in basic and clinical research in the field of serum biomarker discovery, protein-protein interaction and small molecules, such as identification of novel autoantibodies in Behcet disease (Chen et al., 2008) and rheumatoid arthritis. (Sjöberg et al., 2016) Most studies focused on IgG type autoantibodies, we simultaneously tested IgG and IgA autoantibodies in this study considering that IgA joined innate immune defenses and prevented bacterial attachment to the epithelium and regulated bacterial communities in the gut. (Suzuki et al., 2010) We adopted a three-stage strategy combined a proteome-wide screen for novel autoantigens followed by a stringent validation step. We enrolled confirmed IBS patients who fulfilled the Rome III criteria and strictly excluded organic diseases. In addition to healthy controls, we chose STFC, CIPO and IBD which might have similar clinical symptoms and pathophysiology with IBS as disease controls. Finally, the additional large cohorts were used for validation to ensure the accuracy of research. These strategies could enhance the reliability of the results.

To date, there are several antibodies reported in IBS, including antibodies against GnRH, flagellin, food allergens (IgE type), voltage-gated calcium channels (VGCCs), antibodies to cytolethal distending toxin B (anti-CdtB), anti-vinculin, anti-gliadin IgA, anti-enteric neuronal antibodies, antihuman tissue transglutaminase (h-tTG IgA) and deamidated gliadin peptide antibodies (DGP II IgA and DGP II IgG) and anti-alpha3-AChR antibodies. (Törnblom et al., 2007; Fan et al., 2018; Morales et al., 2019) In 2012, Wood et al. found 3 antigens out of an 8,000 immunoproteinarray in the sera of 3 cases of IBS patients, including a nondescript ribonucleoprotein (RNP-complex), a small nuclear ribonuclear polypeptide A, and a Ro-5200 kDa. (Wood et al., 2012) Among the significantly different autoantigens between IBS and HCs in this study, SNRPA was consistent with results of Wood’s. Concerning IBS compared to IBD, IBS-D subjects had higher titers of plasma anti-CdtB (1.49 ± 0.56 vs. 1.04 ± 0.33, *p* < 0.0001) and anti-vinculin (1.66 ± 0.97 vs. 0.88 ± 0.65, *p* = 0.0006) than IBD subjects by ELISA. (Morales et al., 2019) Systemic inflammatory proteins CASP8, AXIN1, ST1A1, and TNFSF14 detected by ProSeek Multiplex Inflammation Kit can distinguish UC patients with IBS. (Moraes et al., 2020) However, these studies mainly focused on previous reported antibodies, systemic inflammatory protein profiles rather than wide screening and the sample sizes were relatively small. We could not differentiate IBS with IBD or STFC in our study which might due to different study design and detection method. We speculated IBS shared similar immunological characteristics with IBD or STFC by HuProt™ microarray*.* In our study, we found diverse autoantibodies (14 IgGs and 14 IgAs) with low positive rates in IBS patients and absence of a specific autoantibody, which indicated autoimmune response targeting a diversity of antigens might involve in the pathogenesis of IBS. This result is consistent with the knowledge -IBS is caused and precipitated by multiple factors with complex mechanisms, and presents as a highly heterogeneous disorder. Considering that multiple rather than specific autoantibodies were found in sera of patients with IBS, a decision tree model demonstrated even combination of multiple autoantibodies have relatively low sensitivity for IBS diagnosis. On the other hand, the positive rate of any IgG/IgA autoantibody in sera of IBS is as higher as 69.0%, with significant difference to HCs (42.7%). Our results are more significant to future IBS pathophysiology research from candidate autoantibodies than clinical use in IBS diagnosis.

Among significantly different autoantibodies we found, anti-ELAVL4 (anti-HuD) is one of the anti-neuronal antibodies. HuD, as an important RNA-binding protein, emerges as a key component in multiple regulatory processes including pre-messenger RNA (mRNA) processing, mRNA stability, and translation and plays important roles in neuronal development and function. (Bronicki and Jasmin, 2013) Anti-HuD had direct excitatory action on visceral sensory and enteric neurons and may be related to antibody-mediated gut dysfunction. (Li et al., 2016) Besides, anti-HuD antibodies could evoke neuronal apoptosis and contribute to enteric nervous system impairment. (De Giorgio et al., 2003) Thus we speculate some IBS patients are related to anti-HuD mediated gut dysmotility. In addition, ANXA1 IgG and IgA was significantly different between IBS and HCs. ANXA1 plays important roles in the innate immune response and promotes resolution of inflammation and wound healing. (Arcone et al., 1993) Moreover, we indeed found PIGP, TCEAL6 and RIOK IgA were related to IBS clinical symptoms. It is necessary to further study the effect of these candidate autoantibodies to enteric neurons and intestinal functions.

There were some limitations of our study. The majority of IBS patients were IBS-D and the number of IBS-C, IBS-M patients were small. We did not enroll IBS-U patients. Besides, the number of CIPO patients was relatively small and we only included STFC and IBD patients as DCs in the second cohort. We used the different scales to evaluate severity of IBS-D (Fan et al., 2017) and IBS-M, IBS-C. (Drossman, 2016) Finally, we failed to purify RIOK1 protein at the last step.

In conclusion, the present study demonstrated multiple rather than specific autoantibodies with low positive rates in sera of IBS compared with HCs, which failed to differentiate IBS with STFC or IBD with solely serological markers. A diversity of autoantibodies existing in IBS indicated extraordinarily complex autoimmune reactions in the pathogenesis. To find the pathogenic autoantigens through autoantibody identification and further study the injury mechanism of autoimmune response are still needed.

## Data Availability

The raw data supporting the conclusion of this article will be made available by the authors, without undue reservation.
