# Supplementary material for: Multiple rather than specific autoantibodies were identified in irritable bowel syndrome with HuProt™ proteome microarray
Source: Front Physiol. 2022 Oct 3;13:1010069. doi: 10.3389/fphys.2022.1010069 (PMC9573966; doi:10.3389/fphys.2022.1010069)
Supplement: Supplementary file 1 [file Table1.docx]

Supplementary Materials

# Supplementary Figure Legends

**Supplementary Figure 1.** Quality control of each microarray by GST immunoblotting (98% of all proteins showed GST signals significantly higher than negative controls). Each microarray contains 24 blocks (A). B showed one of the 24 blocks. The correlation coefficient between duplicate spots for each protein was 0.945 (C). Quality control of IBS focused microarrays determined by GST immunoblotting (D).

**Supplementary Figure 2**. The candidate autoantigens identified in stage I. (A, C) The candidate autoantigens CFAP36 IgG and ACOT7 IgA were specifically identified by sera from the IBS patients, but not from HCs. (B, D) The candidate autoantigens NOL3 IgG and VCY IgA were specifically identified by sera from the IBS patients, but not from DCs. IBS, irritable bowel syndrome; HCs, healthy controls; DCs, disease controls.

# Supplementary Tables

**Supplementary Table 1**. Potential IBS-associated autoantigens identified by HuProt^TM^ human proteome microarray in stage I

| Type | Proteins | IBS vs HCs | | IBS vs DCs | | Mild-moderate vs severe IBS | |
| --- | --- | --- | --- | --- | --- | --- | --- |
|  | (autoantigens) | Fold  change | *P* | Fold  change | *P* | Fold  change | *P* |
| **IgG** | ZNF667 | 2.89 | 0.02 | -- | -- | -- | -- |
|  | CFAP36^#^ | 2.82 | 0.03 | -- | -- | -- | -- |
|  | CFAP36 | 2.69 | 0.02 | -- | -- | -- | -- |
|  | CFAP36 | 2.65 | 0.01 | -- | -- | -- | -- |
|  | TCEAL6^#^ | 2.40 | 0.02 | -- | -- | -- | -- |
|  | TCEAL6 | 2.32 | 0.02 | -- | -- | -- | -- |
|  | DTNBP1 | 2.04 | 0.03 | -- | -- | -- | -- |
|  | APBA1 | 1.92 | 0.04 | -- | -- | -- | -- |
|  | NOL3 | -- | -- | 3.50 | < 0.01 | -- | -- |
|  | HCLS1 | -- | -- | 3.50 | 0.01 | -- | -- |
|  | Nc2b | -- | -- | 3.18 | 0.04 | -- | -- |
|  | TXNDC12 | -- | -- | 3.10 | 0.01 | -- | -- |
|  | ASNA1 | -- | -- | 2.99 | 0.03 | -- | -- |
|  | RTN4 | -- | -- | 2.55 | 0.03 | -- | -- |
|  | FAM46B | -- | -- | 2.21 | 0.04 | -- | -- |
|  | WDR54 | -- | -- | 2.20 | 0.02 | -- | -- |
|  | PCDHGA10 | -- | -- | 2.03 | 0.04 | -- | -- |
|  | PTRH2 | -- | -- | 1.99 | 0.03 | -- | -- |
|  | PAGE5 | -- | -- | 1.95 | 0.04 | -- | -- |
|  | IL1A | -- | -- | 1.94 | 0.04 | -- | -- |
|  | PIGP | -- | -- | 1.92 | 0.04 | -- | -- |
|  | DR1 | -- | -- | -- | -- | 7.50 | 0.03 |
|  | PPM1K | -- | -- | -- | -- | 2.57 | 0.04 |
|  | HNRNPC | -- | -- | -- | -- | 2.29 | 0.04 |
|  | ANKHD1 | -- | -- | -- | -- | 2.13 | 0.04 |
|  | PDPN | -- | -- | -- | -- | 2.00 | 0.03 |
|  | GPBP1_frag | -- | -- | -- | -- | 2.00 | 0.03 |
|  | NASP | -- | -- | -- | -- | 1.98 | 0.04 |
|  | RIOK1 | -- | -- | -- | -- | 1.98 | 0.04 |
|  | LASP1 | -- | -- | -- | -- | 1.95 | 0.01 |
| **IgA** | ACOT7 | 1.86 | 0.04 | -- | -- | -- | -- |
|  | DAB1 | 1.57 | 0.03 | -- | -- | -- | -- |
|  | VCY | -- | -- | 2.78 | < 0.01 | -- | -- |
|  | ANXA1 | -- | -- | 2.65 | 0.03 | -- | -- |
|  | TFAP2E | -- | -- | 2.60 | < 0.01 | -- | -- |
|  | EPS8L1 | -- | -- | 2.50 | < 0.01 | -- | -- |
|  | FGF13 | -- | -- | 2.48 | < 0.01 | -- | -- |
|  | WT1-AS | -- | -- | 2.47 | < 0.01 | -- | -- |
|  | PLEK | -- | -- | 2.45 | 0.03 | -- | -- |
|  | NOP56_frag | -- | -- | 2.44 | 0.03 | -- | -- |
|  | WDR83 | -- | -- | 2.42 | < 0.01 | -- | -- |
|  | NCF1 | -- | -- | 2.40 | < 0.01 | -- | -- |
|  | TRMT2A | -- | -- | 2.38 | < 0.01 | -- | -- |
|  | EXOSC5 | -- | -- | 2.37 | 0.01 | -- | -- |
|  | GLIPR2 | -- | -- | 2.31 | < 0.01 | -- | -- |
|  | PRKCDBP | -- | -- | -- | -- | 2.01 | 0.04 |
|  | WSCD2 | -- | -- | -- | -- | 1.94 | 0.03 |

IBS, irritable bowel syndrome; HCs, healthy controls; DCs, disease controls. #Isotype of the same protein. --none significance.

**Supplementary Table 2**. The detailed information of the significantly different proteins

| Protein name | Protein ID | Protein description | Functions |
| --- | --- | --- | --- |
| RIOK1 | Q9BRS2 | Serine/threonine-protein kinase RIO1 | Involved in the final steps of cytoplasmic maturation of the 40S ribosomal subunit. Involved in processing of 18S-E pre-rRNA to the mature 18S rRNA. |
| ANXA1 | P04083 | Annexin A1 | Plays important roles in the innate immune response as effector of glucocorticoid-mediated responses and regulator of the inflammatory process. Has anti-inflammatory activity. |
| ELAVL4 | P26378 | ELAV-like protein 4 | RNA-binding protein. Plays a role in neuron-specific RNA processing by stabilization of mRNAs of neuronal proteins, contributing to the differentiation of neural progenitor cells, nervous system development, learning and memory mechanisms. |
| EXOSC5 | Q9NQT4 | Exosome complex component RRP46 | Participates in cellular RNA processing, degradation events and apoptotic DNA degradation. |
| FAM46B | Q96A09 | Terminal nucleotidyltransferase 5B | Probable nucleotidyltransferase that may act as a non-canonical poly(A) RNA polymerase. |
| APBA1 | Q02410 | Amyloid-beta A4 precursor protein-binding family A member 1 | Putative function in synaptic vesicle exocytosis by binding to Munc18-1, an essential component of the synaptic vesicle exocytotic machinery. |
| WDR83 | Q9BRX9 | WD repeat domain-containing protein 83 | Molecular scaffold protein for various multimeric protein complexes. Acts as a module in the assembly of a multicomponent scaffold for the ERK pathway. |
| WT1-AS | Q06250 | Putative Wilms tumor upstream neighbor 1 gene protein | Promotes cell apoptosis in hepatocellular carcinoma. |
| PRKCDBP | Q969G5 | Caveolae-associated protein 3 | Plays a role in caveola formation in a tissue-specific manner. |
| DAB1 | O75553 | Disabled homolog 1 | Adapter molecule functioning in neural development. |
| TRMT2A | Q8IZ69 | tRNA (uracil-5-)-methyltransferase homolog A | May be involved in nucleic acid metabolism and/or modifications. |
| SNRPA | P09012 | U1 small nuclear ribonucleoprotein A | Component of the spliceosomal U1 snRNP, which is essential for recognition of the pre-mRNA 5' splice-site and the subsequent assembly of the spliceosome. |
| TFAP2E | Q6VUC0 | Transcription factor AP-2-epsilon | Sequence-specific DNA-binding protein that interacts with inducible viral and cellular enhancer elements to regulate transcription of selected genes. |
| WDR54 | Q9H977 | WD repeat-containing protein 54 | Has a regulatory effect on ERK signaling pathway activity in response to EGF stimulation. |
| PIGP | P57054 | Phosphatidylinositol N-acetylglucosaminyltransferase subunit P | Part of the complex catalyzing the transfer of N-acetylglucosamine from UDP-N-acetylglucosamine to phosphatidylinositol, the first step of GPI biosynthesis. |
| CACNG3 | O60359 | Voltage-dependent calcium channel gamma-3 subunit | Regulates the trafficking to the somatodendritic compartment and gating properties of AMPA-selective glutamate receptors. |
| VCY | O14598 | Testis-specific basic protein Y | May mediate a process in spermatogenesis or may play a role in sex ratio distortion. |
| PAGE5 | Q96GU1 | P antigen family member 5 | - |
| PPM1K | Q8N3J5 | Protein phosphatase 1K, mitochondrial | Regulates the mitochondrial permeability transition pore and is essential for cellular survival and development. |
| GPBP1_frag | Q86WP2 | Vasculin | Functions as a GC-rich promoter-specific transactivating transcription factor. |
| HCLS1 | P14317 | Hematopoietic lineage cell-specific protein | Plays a role in antigen receptor signaling for both clonal expansion and deletion in lymphoid cells. |
| ZNF667 | Q5HYK9 | Zinc finger protein 667 | May be involved in transcriptional regulation. |
| TCEAL6 | Q6IPX3 | Transcription elongation factor A protein-like 6 | May be involved in transcriptional regulation. |
| PTRH2 | Q9Y3E5 | Peptidyl-tRNA hydrolase 2, mitochondrial | Promotes caspase-independent apoptosis by regulating the function of two transcriptional regulators, AES and TLE1. |
