# Supplementary figures and images for: Multiple rather than specific autoantibodies were identified in irritable bowel syndrome with HuProt™ proteome microarray
Source: Front Physiol. 2022 Oct 3;13:1010069. doi: 10.3389/fphys.2022.1010069 (PMC9573966; doi:10.3389/fphys.2022.1010069)

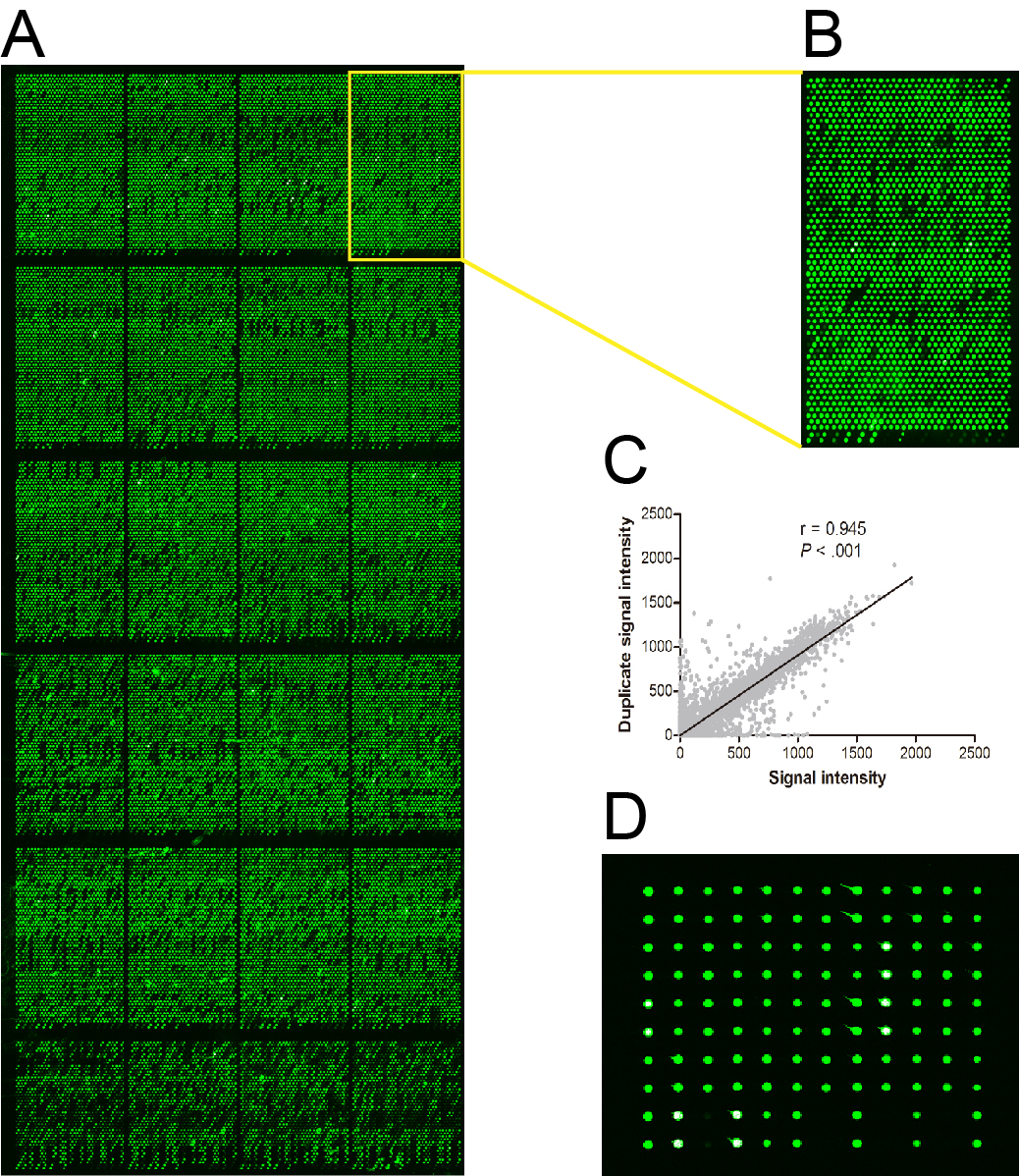

Supplement: Supplementary file 2 [file Image1.JPEG]

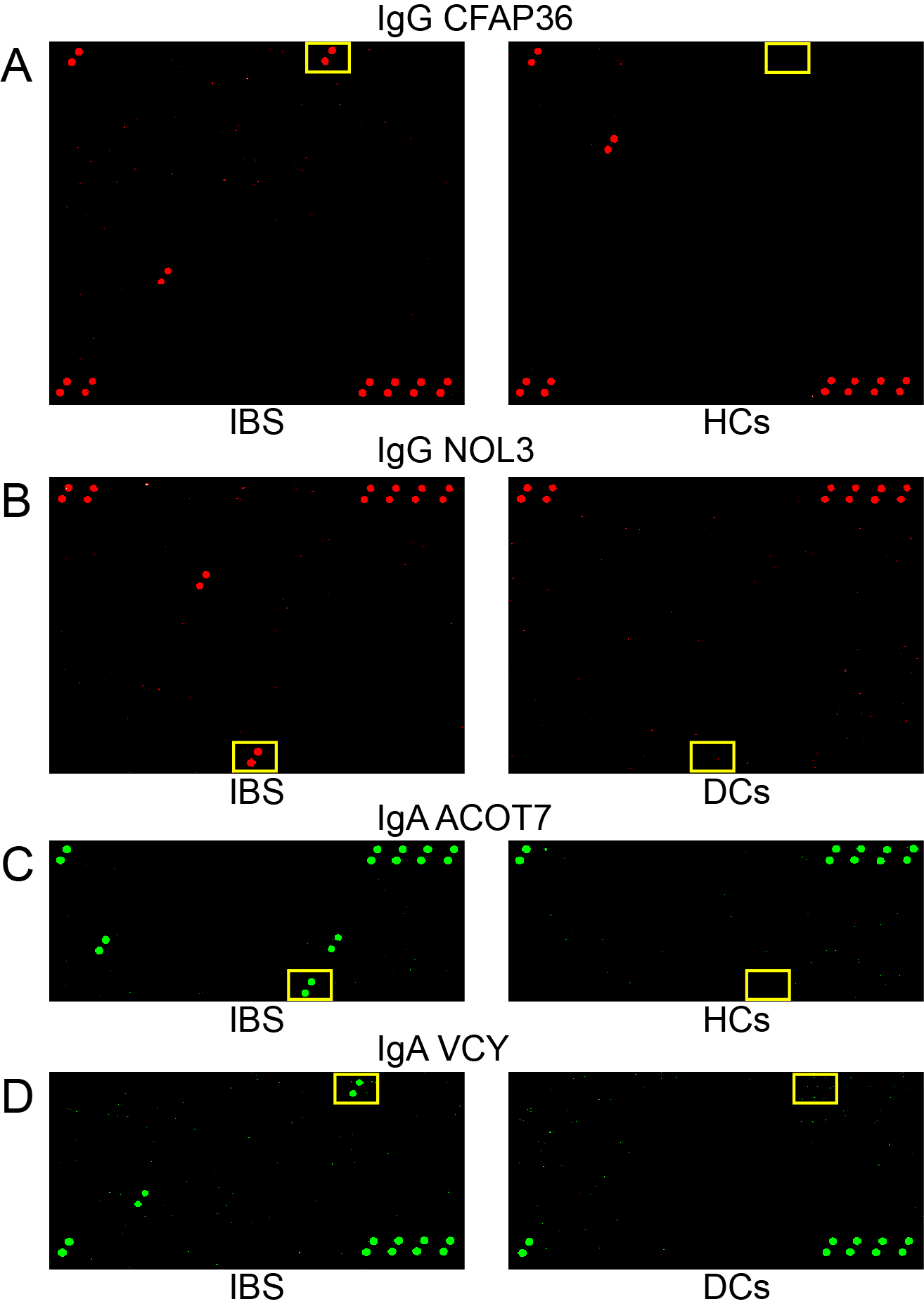

Supplement: Supplementary file 3 [file Image2.JPEG]
